# Microbiota populations in supragingival plaque, subgingival plaque, and saliva habitats of adult dogs

**DOI:** 10.1186/s42523-021-00100-9

**Published:** 2021-05-17

**Authors:** Patrícia M. Oba, Meredith Q. Carroll, Celeste Alexander, Helen Valentine, Amy J. Somrak, Stephanie C. J. Keating, Adrianna M. Sage, Kelly S. Swanson

**Affiliations:** 1 Department of Animal Sciences, University of Illinois at Urbana-Champaign, 1207 West Gregory Drive, 162 Animal Sciences Laboratory, IL 61801 Urbana, USA; 2 Division of Nutritional Sciences, University of Illinois at Urbana-Champaign, 61801 Urbana, IL USA; 3 Division of Animal Resources, University of Illinois at Urbana-Champaign, 61801 Urbana, IL USA; 4 Department of Veterinary Clinical Medicine, College of Veterinary Medicine, University of Illinois, 61801 Urbana, IL USA; 5 Department of Nutrition, Dominican University, 60305 River Forest, IL USA

**Keywords:** Canine, Oral microbiome, Periodontal disease, 16S rRNA gene

## Abstract

**Background:**

Oral diseases are common in dogs, with microbiota playing a prominent role in the disease process. Oral cavity habitats harbor unique microbiota populations that have relevance to health and disease. Despite their importance, the canine oral cavity microbial habitats have been poorly studied. The objectives of this study were to (1) characterize the oral microbiota of different habitats of dogs and (2) correlate oral health scores with bacterial taxa and identify what sites may be good options for understanding the role of microbiota in oral diseases. We used next-generation sequencing to characterize the salivary (SAL), subgingival (SUB), and supragingival (SUP) microbial habitats of 26 healthy adult female Beagle dogs (4.0 ± 1.2 year old) and identify taxa associated with periodontal disease indices.

**Results:**

Bacterial species richness was highest for SAL, moderate for SUB, and lowest for SUP samples (*p* < 0.001). Unweighted and weighted principal coordinates plots showed clustering by habitat, with SAL and SUP samples being the most different from one another. Bacteroidetes, Proteobacteria, Firmicutes, Fusobacteria, Actinobacteria, and Spirochaetes were the predominant phyla in all habitats. *Paludibacter*, *Filifactor, Peptostreptococcus, Fusibacter, Anaerovorax, Fusobacterium, Leptotrichia, Desulfomicrobium*, and *TG5* were enriched in SUB samples, while *Actinomyces*, *Corynebacterium*, *Leucobacter*, *Euzebya*, *Capnocytophaga*, *Bergeyella*, *Lautropia, Lampropedia, Desulfobulbus, Enhydrobacter*, and *Moraxella* were enriched in SUP samples. *Prevotella, SHD-231, Helcococcus, Treponema*, and *Acholeplasma* were enriched in SAL samples. *p-75-a5*, *Arcobacter*, and *Pasteurell*a were diminished in SUB samples. *Porphyromonas*, *Peptococcus*, *Parvimonas*, and *Campylobacter* were diminished in SUP samples, while *Tannerella*, *Proteocalla*, *Schwartzia*, and *Neisseria* were diminished in SAL samples. A*ctinomyces, Corynebacterium, Capnocytophaga*, *Leptotrichia*, and *Neisseria* were associated with higher oral health scores (worsened health) in plaque samples.

**Conclusions:**

Our results demonstrate the differences that exist among canine salivary, subgingival plaque and supragingival plaque habitats. Salivary samples do not require sedation and are easy to collect, but do not accurately represent the plaque populations that are most important to oral disease. Plaque *Actinomyces*, *Corynebacterium*, *Capnocytophaga*, *Leptotrichia*, and *Neisseria* were associated with higher (worse) oral health scores. Future studies analyzing samples from progressive disease stages are needed to validate these results and understand the role of bacteria in periodontal disease development.

## Background

Periodontal diseases are very common in dogs, with 44–64 % of dogs being affected by the disease [1–4]. Oral microbiota plays a prominent role in periodontal disease pathogenesis [5–8], as it develops as a result of plaque build-up on the teeth [9–13]. The calcification of the plaque forms the oral calculus, and the porous surface of the calculus provides the perfect condition for bacterial colonization and proliferation, which can lead to damage to the periodontium by causing deterioration of gingival connective tissue [14]. In healthy dogs, the gram-negative bacteria species are believed to be predominant, while gram-positive anaerobic species predominate in diseased animals [9].

The information generated by bacteriological analysis of the oral cavity is highly dependent on the location, or habitat, in the mouth as well as sampling technique [15]. In humans, bacterial communities were identified in seven different oral cavity habitats [buccal mucosa, keratinized gingiva, hard palate; saliva, tongue, subgingival (SUB), and supragingival (SUP) plaques], with tooth-associated communities being distinct from the other oral habitats [16]. Although the technologies used were limited and the number of animals was low, similar studies have been performed in dogs. In one study, bacterial communities were identified in five different oral cavity habitats (SUB and SUP plaque, tongue, tonsils, and cheek mucosa) of seven Beagle dogs [17]. In that study, microbial communities colonizing the tooth-associated habitats of the oral cavity were quite different from those colonizing the soft tissues. Furthermore, the microbiota populations most relevant to disease were in the SUB (below the gum line) and SUP (above the gum line) plaque biofilms [17]. In another study, 14 Labrador retrievers were used to identify the microbiota from four different niches within the canine oral cavity (SUP plaque, saliva, buccal and tongue dorsum mucosa) and reported that saliva exhibited the highest variability in microbial composition among dogs, yet the lowest bacterial diversity amongst all niches [18]. Local factors such as oxygen tension, pH, and mucosal surfaces may impact the local microbiota and may be a reason for these differences. Bacterial communities may also differ due to the surface type, including soft tissue surfaces (buccal and tongue dorsum mucosa), hard tissue surfaces (SUP plaque), and saliva. Bacterial groups or metabolites, either on their own or in combinations (i.e., signatures), may be valuable in diagnosing and/or monitoring companion animal periodontal diseases in the future. Microbiota signatures are greatly dependent on habitat, however, so it is important to identify the individual or groups of bacteria that best serve as disease biomarkers and may be used for this purpose [18].

The sampling type is also important. For SUB biofilms, the information gathered from curette samples frequently differs from that obtained from paper-point samples. This is thought to be different because curettes collect plaque from the entire pocket, whereas the plaque adsorbed onto a paper point is derived mostly from the outer layers of the biofilm, which might contain the more pathogenic microbiota [19–21]. Additionally, paper points are less successful at collecting plaque in apical portions of a pocket than from areas near the gingival margin [22]. Furthermore, bacteria in SUB biofilms are not homogeneously distributed within the pocket, and for that reason, paper‐point samples might not accurately represent the microbiota population at the base of the pocket where the disease is progressing [15].

Because a majority of microbial species cannot be cultured [15], advances in disease prevention have been limited. DNA-based methods of describing and studying microbes have many advantages, including the fact that they do not rely on culture methods, have greater precision, and are more accurate than traditional culture methods. Moreover, next-generation techniques that have greater speed and lower costs than traditional sequencing methods are now available. These methods allow the complete characterization of microbial populations, including those in the oral cavity, a niche that has been poorly studied in dogs. Most of the studies conducted in the past used culture techniques [11, 14, 23] or checkerboard DNA–DNA hybridization [17] that analyzed a selected group of bacteria. The majority of studies also only collected samples from one site or compared the difference between SUP and SUB plaque.

Characterization of the canine oral microbiome, including saliva and plaque habitats, using next-generation sequencing methods may not only identify the species present (phylogeny) but also highlight metabolic and biological pathways contributing to physiologic outcomes (metagenomics data). Once the oral microbiome of healthy dogs has been characterized, future clinical experiments focused on periodontal disease patients may be performed. Additionally, the use of oral swabs to evaluate the oral microbiota from dogs is a comparatively simple procedure when compared to the collection of canine dental plaque. Because it is less invasive and easy to collect, it is important to determine how well the salivary microbiota community relates to that of plaque. Similarities and/or differences among those populations will help determine whether saliva sampling can be used as a proxy for the characterization of plaque. Even though the dogs used in this study did not have severe periodontal disease, there was significant variability in regard to oral health scores. Therefore, our results will contribute to the foundation in the oral health area and provide guidance for future studies focused on periodontal disease of dogs.

## Results

### Dental scoring and salivary pH

 First, gingivitis, plaque, calculus and pocket scores were conducted by a board-certified veterinary dentist and salivary pH was measured using pH strips. All dental scores and salivary pH are presented in Table 1. The average tooth pocket depth of dogs was normal (1.6 ± 0.4 mm) and pocket bleeding was minor (1.5 ± 1.4), resulting in an average final pocket score of 2.8 ± 2.9. Dogs had extensive plaque coverage (3.6 ± 0.4) and thickness (2.7 ± 0.3), resulting in a final plaque score of 9.7 ± 2.1 [0 (low) to 12 (maximum)]. Dogs had moderate calculus coverage (2.8 ± 0.8) and thickness (2.0 ± 0.6), resulting in a final calculus score of 6.1 ± 3.0 [0 (low) to 12 (maximum)]. Dogs had mild gingivitis (1.0 ± 0.8) and an overall oral health score (OHS; sum of plaque, calculus, gingivitis, and pocket scores) of 19.6 ± 7.4. Finally, mean salivary pH was 7.96 ± 0.51.

**Table 1:** Dental scoring and salivary pH from healthy adult dogs (*n* = 26)

Item	Mean	Std Deviation
Pocket Depth	1.6	0.41
Pocket Bleeding	1.5	1.44
Plaque Coverage	3.6	0.44
Plaque Thickness	2.7	0.33
Calculus Coverage	2.8	0.85
Calculus Thickness	2.0	0.58
Oral Health Score	19.6	7.43
Plaque Score	9.7	2.10
Calculus Score	6.1	2.95
Gingivitis Score	1.0	0.76
Pocket Score	2.8	2.94
Salivary pH	7.96	0.506

### Canine oral microbiome composition

**Fig. 1:**
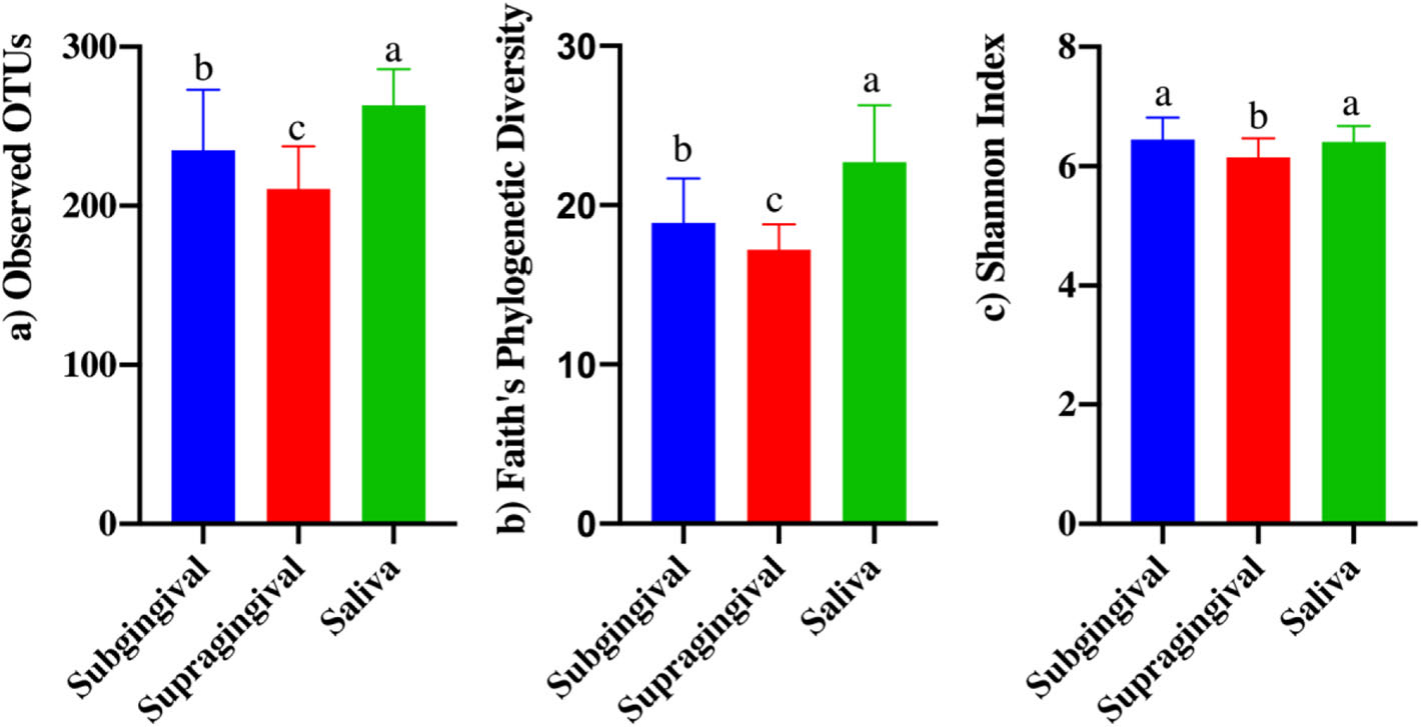
Bacterial alpha diversity indices of canine salivary and plaque samples as assessed by the observed operational taxonomic units (OTU) (**a**), Faith’s Phylogenetic Diversity (**b**), and Shannon Index (**c**). Groups with different superscripts differ (*p*<0.001)

**Fig. 2:**
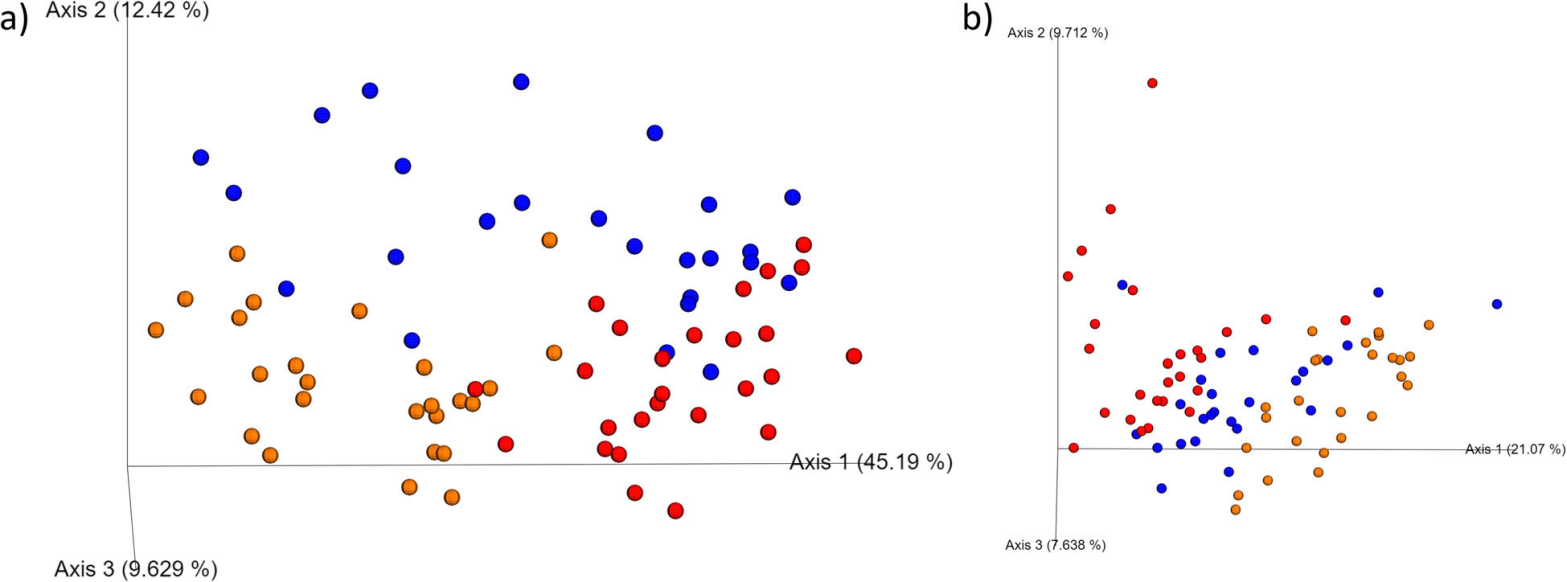
Principal coordinates analysis (PCoA) plots of weighted (**a**) and unweighted (**b**) UniFrac distances of oral microbial communities performed on the 97% OTU abundance matrix using QIIME

 After pH was measured and teeth were scored, SUB plaque, SUP plaque, and saliva samples were collected for microbiota analysis so the communities of each habitat could be characterized and compared. Illumina sequencing produced a total of 3,897,739 16S rRNA amplicon sequences, with an average of 50,620 sequences per sample after quality filtering. Analyses were conducted with all samples rarified to a level of 19,981 sequences. Alpha and beta diversity indices were affected by sample type (habitat). Species richness differed among habitats, with SAL having the highest observed OTU (Fig. 1a) and Faith’s phylogenetic diversity (Fig. 1b), SUB having moderate richness, and SUP having the lowest richness (*p* < 0.0001). SAL and SUB had similar Shannon index values that were higher than that of SUP (Fig. 1c, *p* < 0.0001). Weighted (Fig. 2a) and unweighted (Fig. 2b) PCoA plots showed how the samples clustered according to oral habitat (*p* < 0.001). In both plots, the SAL and SUP clusters were nearly completely separated, with the SUB having overlap with each.

**Fig. 3:**
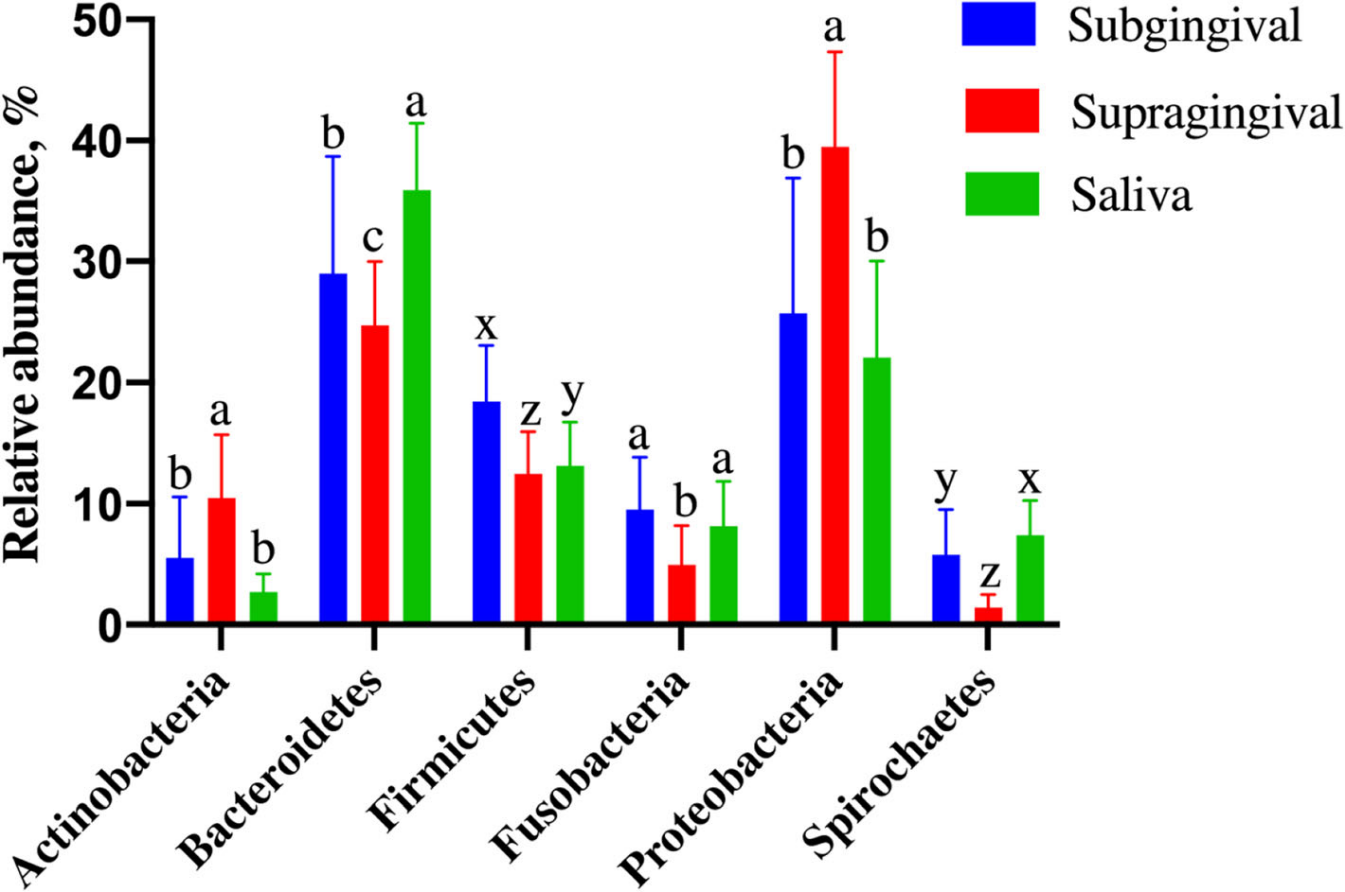
Predominant oral bacterial phyla (relative abundance, %) present in plaque (supragingival and subgingival plaque) and saliva samples of healthy adult dogs. ^a-c^Means with different superscripts within phyla differ by Tukey's test (*p*<0.05). ^w-z^Means with different superscripts within phyla differ by Wilcoxon's test (*p*<0.05)

The oral microbiota measured in this study contained a diverse array of bacteria, including the detection of 13 phyla, with three phyla (Bacteriodetes, Proteobacteria, Firmicutes) accounting for more than 70 % of sequences (Fig. 3). Oral habitat had a significant influence on the bacterial phyla (Fig. 3; Table 2), with SAL and SUB samples having lower Actinobacteria, GN02, and Proteobacteria relative abundances than SUP samples (*p* < 0.0001). SAL and SUB samples had higher Euryarchaeota and Fusobacteria relative abundances than SUP samples (*p* < 0.0001). SUB and SUP samples had lower Chloroflexi (*p* = 0.0016) and ZB3 (*p* = 0.0002) relative abundances than SAL samples. Bacteroidetes, Spirochaetes, and Tenericutes relative abundances were highest in SAL, followed by SUB, and lowest in SUP samples (*p* < 0.0001). Firmicutes relative abundance was higher in SUB, followed by SAL, and lowest in SUP samples (*p* < 0.0001). SR1 relative abundance was higher in SAL, followed by SUP, and lowest in SUB samples (*p* < 0.0001). Synergistetes relative abundance was higher in SUB, followed by SUP, and lowest in SAL samples (*p* < 0.0001). TM7 relative abundance was higher in SAL than SUB samples (*p* = 0.0026).

**Table 2:** Oral bacterial phyla (relative abundance, %) present in plaque (supragingival and subgingival plaque) and saliva samples from healthy adult dogs

	Source			Statistics	
Phyla	Saliva (*n* = 26)	Subgingival (*n* = 25)	Supragingival (*n* = 26)	SEM	*P*-values
Euryarchaeota	0.3^a^	0.3^a^	0.1^b^	0.068	0.0351
Actinobacteria	2.7^b^	5.5^b^	10.5^a^	0.838	< 0.0001
Bacteroidetes	35.9^a^	29.0^b^	24.7^c^	1.386	< 0.0001
Chlorobi	1.2	1.4	1.3	0.147	0.8963
Chloroflexi	0.9^a^	0.3^b^	0.4^b^	0.139	0.0016
Elusimicrobia	0.03	0.04	0.03	0.011	0.0529
Firmicutes	13.2^y^	18.5^x^	12.5^z^	0.770	< 0.0001
Fusobacteria	8.1^a^	9.5^a^	4.9^b^	0.746	< 0.0001
GN02	0.2^b^	0.3^b^	0.6^a^	0.082	< 0.0001
Proteobacteria	22.1^b^	25.7^b^	39.5^a^	1.788	< 0.0001
SR1	4.2^a^	2.0^c^	3.1^b^	0.338	< 0.0001
Spirochaetes	7.4^x^	5.8^y^	1.4^z^	0.547	< 0.0001
Synergistetes	0.3^z^	1.1^x^	0.6^y^	0.100	< 0.0001
TM7	0.08^a^	0.04^b^	0.05^ab^	0.010	0.0026
Tenericutes	3.3^a^	0.5^b^	0.2^c^	0.226	< 0.0001
WPS-2	0.06	0.04	0.01	0.011	0.3538
ZB3	0.07^a^	0.02^b^	0.01^b^	0.010	0.0002

^a−c^Means with different superscripts within a row differ by Tukey’s test (*p* < 0.05)

^w−z^Means with different superscripts within a row differ by Wilcoxon’s test (*p* < 0.05)

**Fig. 4:**
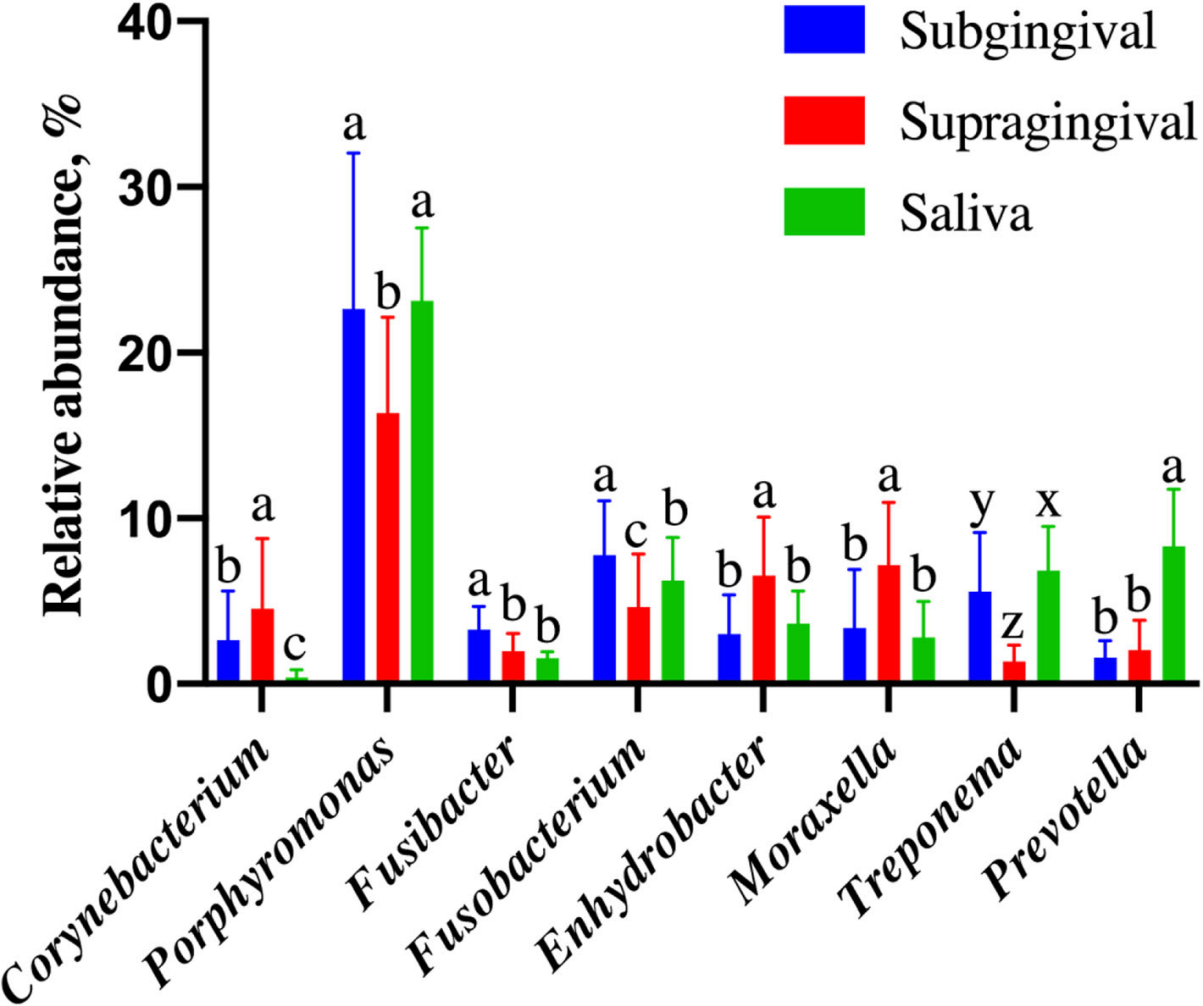
Predominant oral bacterial genera (relative abundance, %) present in plaque (supragingival and subgingival plaque) and saliva samples from healthy adult dogs. ^a-c^Means with different superscripts within a genera differ by Tukey's test (*p*<0.05). ^w-z^Means with different superscripts within a genera differ by Wilcoxon's test (*p*<0.05)

The most predominant genera in SAL and SUB samples were *Porphyromonas* and *Fusobacterium*. In SUP samples, *Porphyromonas*, *Moraxella*, and *Fusobacterium* were predominant, making up to approximately 30 % of the bacteria present (Fig. 4). Oral habitat had a significant influence on the oral bacterial genera (Fig. 4; Table 3). SAL and SUB samples had lower relative abundances of *Actinomyces*, *Leucobacter*, *Lautropia*, *Lampropedia*, *Enhydrobacter*, and *Moraxella* than SUP samples (*p* < 0.0001). SAL and SUB samples had higher relative abundances of *Porphyromonas*, *Peptococcus*, *Parvimonas*, and *Campylobacter* than SUP samples (*p* < 0.0001). SUB and SUP samples had lower relative abundances of *SHD-231*, *Prevotella*, and *Helcococcus* than SAL (*p* < 0.0001). SUB and SUP samples had higher relative abundances of *Tannerella, Proteocatella, Schwartzia*, and *Neisseria* than SAL (*p* < 0.0001). SAL and SUP samples had higher relative abundances of *Arcobacter, p-75-a5*, and *Pasteurella* than SUB samples (*p* = 0.003). SAL and SUP samples had lower relative abundances of *Filifactor, Fusibacter*, and *Leptotrichia* than SUB samples (*p* < 0.0001). *Corynebacterium*, *Euzebya*, *Capnocytophaga*, *Bergeyella*, and *Desulfobulbus* relative abundances were highest in SUP, followed by SUB, and lowest in SAL samples (*p* < 0.0001). *Anaerovorax, Desulfomicrobium*, and *TG5* relative abundances were highest in SUB, followed by SUP, and lowest in SAL samples (*p* < 0.0001). *Acholeplasma* and *Treponema* relative abundances were highest in SAL, followed by SUB, and lowest in SUP samples (*p* < 0.0001). *Bacteroides* and *Parabacteroides* relative abundances were highest in SAL samples than SUB samples (*p* = 0.0453). *Peptostreptococcus* (*p* < 0.0001) and *Fusobacterium* (*p* = 0.0002) relative abundances were highest in SUB samples, followed by SAL, and lowest in SUP samples. *Oscillospira* relative abundance was higher in SUP samples than SUB samples (*p* = 0.01). *Propionivibrio* relative abundance was higher in SUP samples than SAL samples (*p* = 0.005).

**Table 3:** Oral bacterial genera (relative abundance, %) present in plaque (supragingival and subgingival plaque) and saliva samples from healthy adult dogs

	Source			Statistics	
Genera	Saliva (*n* = 26)	Subgingival (*n* = 25)	Supragingival (*n* = 26)	SEM	*P*-values
*Actinomyces*	1.8^b^	1.8^b^	3.1^a^	0.245	< 0.0001
*Corynebacterium*	0.4^c^	2.6^b^	4.5^a^	0.590	< 0.0001
*Leucobacter*	0.1^b^	0.4^b^	1.6^a^	0.155	< 0.0001
*Euzebya*	0.3^z^	0.5^y^	0.6^x^	0.069	0.0008
*Bacteroides*	1.0^a^	0.7^b^	1.1^ab^	0.137	0.0453
*Paludibacter*	0.1^c^	0.5^a^	0.3^b^	0.053	< 0.0001
*Parabacteroides*	0.3^a^	0.1^b^	0.2^ab^	0.040	0.0015
*Porphyromonas*	23.1^a^	22.6^a^	16.4^b^	1.341	< 0.0001
*Tannerella*	0.3^b^	0.6^a^	0.5^a^	0.039	< 0.0001
*Prevotella*	8.3^a^	1.5^b^	2.1^b^	0.459	< 0.0001
*Capnocytophaga*	0.2^c^	0.5^b^	0.9^a^	0.099	< 0.0001
*Bergeyella*	0.3^c^	0.5^b^	2.0^a^	0.204	< 0.0001
*SHD-231*	0.9^a^	0.3^b^	0.4^b^	0.139	0.0016
*Streptococcus*	0.2	0.1	0.2	0.070	0.2643
*Clostridium*	0.1	0.1	0.1	0.017	0.6114
*Catonella*	0.4	0.5	0.5	0.052	0.7442
*Peptococcus*	1.3^a^	2.3^a^	0.6^b^	0.239	< 0.0001
*Filifactor*	0.6^b^	1.3^a^	0.7^b^	0.102	< 0.0001
*Peptostreptococcus*	0.2^b^	0.4^a^	0.1^c^	0.059	< 0.0001
*Proteocatella*	0.1^b^	0.8^a^	0.5^a^	0.111	< 0.0001
*Oscillospira*	0.4^ab^	0.5^a^	0.1^b^	0.096	0.0094
*Schwartzia*	0.04^b^	0.16^a^	0.06^a^	0.019	< 0.0001
*Fusibacter*	1.6^b^	3.3^a^	2.0^b^	0.205	< 0.0001
*Anaerovorax*	0.1^z^	0.3^x^	0.2^y^	0.033	< 0.0001
*Helcococcus*	0.2^a^	0.1^b^	0.1^b^	0.031	< 0.0001
*Parvimonas*	0.5^a^	0.5^a^	0.2^b^	0.098	< 0.0001
*p-75-a5*	0.2^a^	0.1^b^	0.2^a^	0.029	< 0.0001
*Fusobacterium*	6.3^b^	7.7^a^	4.6^c^	0.599	0.0002
*Leptotrichia*	0.04^b^	0.95^a^	0.17^b^	0.139	< 0.0001
*Lautropia*	0.1^b^	0.2^b^	0.7^a^	0.107	< 0.0001
*Lampropedia*	0.5^b^	0.7^b^	2.4^a^	0.201	< 0.0001
*Neisseria*	0.8^b^	2.3^a^	2.5^a^	0.332	< 0.0001
*Propionivibrio*	0.1^b^	0.1^ab^	0.2^a^	0.021	0.0049
*Desulfobulbus*	0.1^c^	0.5^b^	1.6^a^	0.197	< 0.0001
*Desulfomicrobium*	0.7^z^	2.5^x^	1.5^y^	0.213	< 0.0001
*Desulfovibrio*	0.3	0.4	0.5	0.094	0.2526
*Arcobacter*	2.8^a^	1.2^b^	2.4^a^	0.560	0.0031
*Campylobacter*	2.5^a^	2.5^a^	1.5^b^	0.186	< 0.0001
*Wolinella*	0.8	0.7	0.4	0.130	0.2005
*Pasteurella*	0.7^a^	0.4^b^	0.8^a^	0.093	< 0.0001
*Enhydrobacter*	3.7^b^	3.0^b^	6.5^a^	0.536	< 0.0001
*Moraxella*	2.8^b^	3.3^b^	7.2^a^	0.637	< 0.0001
*Treponema*	6.9^x^	5.6^y^	1.3^z^	0.517	< 0.0001
*TG5*	0.3^z^	1.1^x^	0.6^y^	0.110	< 0.0001
*Acholeplasma*	2.6^a^	0.2^b^	0.1^c^	0.186	< 0.0001

^a−c^Means with different superscripts within a row differ by Tukey’s test (*p* < 0.05)

^w−z^Means with different superscripts within a row differ by Wilcoxon’s test ( < 0.05)

**Fig. 5:**
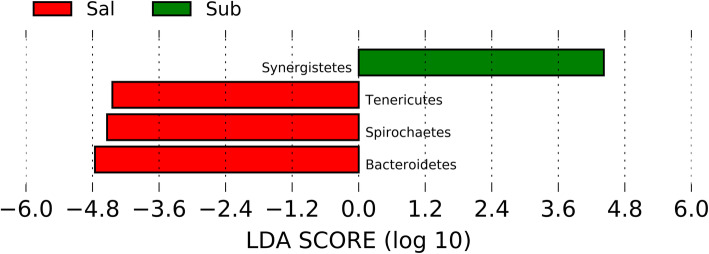
LEfSe results of oral samples identified bacterial phyla enriched in salivary (SAL) and subgingival (SUB) plaque of healthy adult dogs. LDA score ≥ 3

**Fig. 6:**
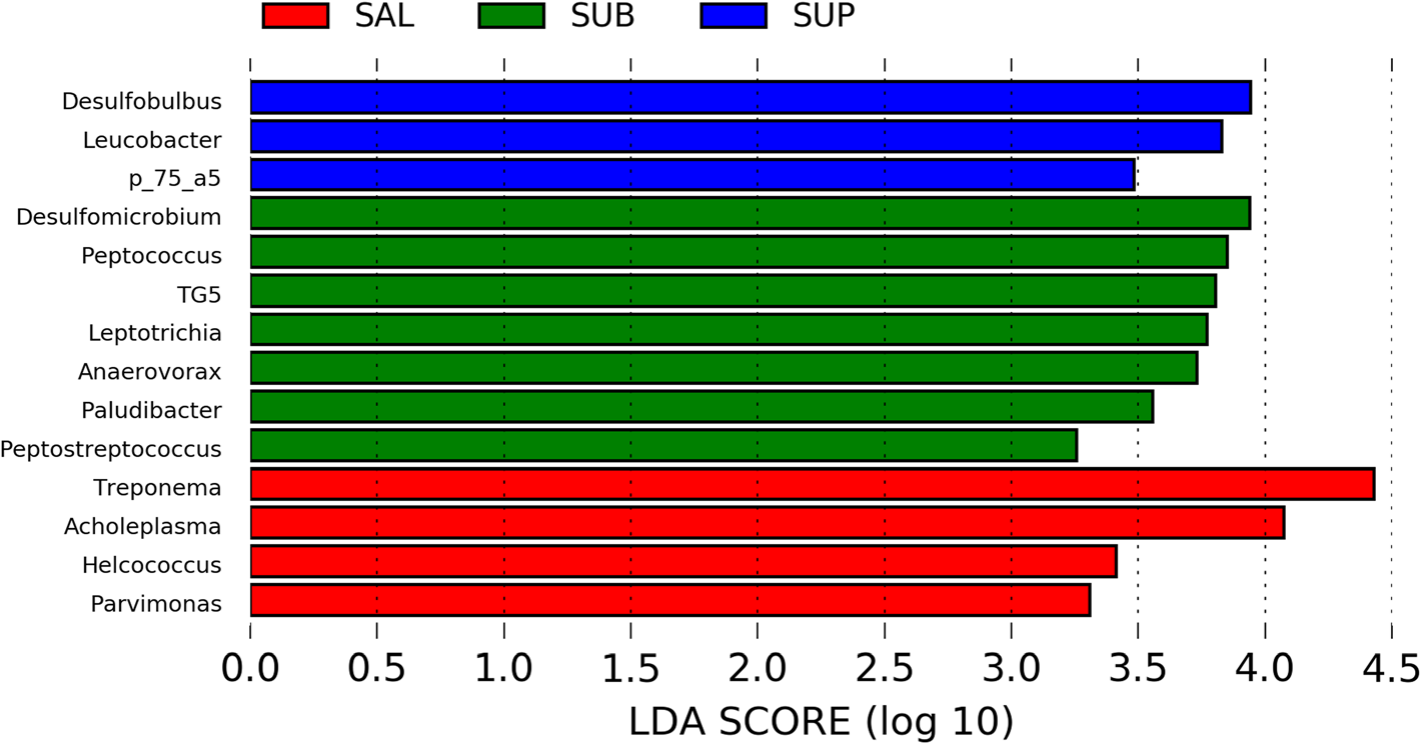
LEfSe results of oral samples identified bacterial genera enriched in salivary (SAL), subgingival (SUB) plaque, and supragingival (SUP) plaque of healthy adult dogs. LDA score ≥ 3

Linear discriminant analysis effect size (LEfSe) identified 4 phyla and 14 genera that were enriched in the three distinct habitats [linear discriminant analysis (LDA) ≥ 3]. One phylum was enriched in SUB plaque (Synergistetes) and 3 phyla were enriched in SAL (Tenericutes, Spirochaetes, and Bacteroidetes) (Fig. 5). Three genera were enriched in SUP plaque (*Desulfobulbus*, *Leucobacter*, and *p_75_a5*), 7 genera were enriched in SUB plaque (*Desulfomicrobium*, *Peptooccus*, *Leptotrichia*, *Anaerovorax*, *TG5*, *Paludibacter*, and *Peptostreptococcus*), and 4 genera were enriched in SAL (*Treponema*, *Acholeplasma*, *Helcococcus*, and *Parvimonas*) (Fig. 6).

### Correlation of canine oral microbiome composition and oral scores and salivary pH

To increase our understanding of the role that microbiota play in oral health and disease, bacterial taxa were correlated with gingivitis, plaque, calculus, and pocket scores, OHS, and salivary pH. Several bacterial phyla and genera were significantly correlated with OHS and salivary pH (Tables 4, 5, 6, 7, 8 and 9). Relative abundance of Actinobacteria was positively correlated with OHS (worsened health), gingivitis score, pocket score, and salivary pH in plaque samples (SUB and SUP). Relative abundance of Proteobacteria was positively correlated with all components of the OHS and salivary pH in SUP samples, and positively correlated with all oral scores except for plaque score and salivary pH in SUB samples. Relative abundance of Bacteroidetes was negatively correlated with all components of the OHS and salivary pH in SUP samples, and with all the oral scores except for plaque score and salivary pH in SUB samples. Relative abundance of Firmicutes was negatively correlated with all oral scores except for plaque score and salivary pH in SUB samples, with pocket and calculus score, and salivary pH in SUP samples, and with pocket and gingivitis score in SAL samples. Relative abundance of SR1 was negatively correlated with all components of OHS and salivary pH in plaque samples. Relative abundance of Tenericutes was negatively correlated with OHS, gingivitis score, pocket score, and salivary pH in SUP samples, and with pocket score, gingivitis score, and salivary pH in SUB samples, and with OHS and salivary pH in SAL samples.

**Table 4:** Correlation coefficients (r) between oral health scores, plaque scores, calculus scores, gingivitis scores, pocket scores, pH, and bacterial phyla in subgingival samples (*n* = 25)^1^

Phyla	Salivary pH		Pocket Score		Gingivitis Score		Calculus Score		Plaque Score		Oral Health Score	
	r	p	r	p	r	p	r	p	r	p	r	p
Actinobacteria	**0.60**	**0.0017**	**0.78**	**< 0.0001**	**0.70**	**< 0.0001**	0.37	0.0666	0.33	0.1129	**0.63**	**0.0008**
Bacteroidetes	**-0.67**	**0.0002**	**-0.82**	**< 0.0001**	**-0.78**	**< 0.0001**	**-0.51**	**0.0100**	-0.39	0.0552	**-0.72**	**< 0.0001**
Chlorobi	-0.08	0.7181	**-0.47**	**0.0183**	**-0.47**	0.0181	-0.22	0.2806	-0.24	0.239	-0.40	0.0503
Firmicutes	**-0.69**	**0.0001**	**-0.85**	**< 0.0001**	**-0.81**	**< 0.0001**	**-0.54**	**0.0050**	-0.37	0.0688	**-0.75**	**< 0.0001**
Fusobacteria	0.28	0.1692	**0.47**	**0.0181**	**0.58**	**0.0025**	0.27	0.1943	0.20	0.3467	**0.41**	**0.0413**
GN02	0.22	0.2835	**0.50**	**0.0118**	0.32	0.1230	0.19	0.3642	0.35	0.0879	**0.41**	**0.0437**
Proteobacteria	**0.69**	**0.0001**	**0.86**	**< 0.0001**	**0.81**	**< 0.0001**	**0.54**	**0.0054**	0.34	0.0928	**0.74**	**< 0.0001**
SR1	**-0.40**	**0.0477**	**-0.47**	**0.0191**	**-0.61**	**0.0013**	**-0.66**	**0.0003**	**-0.47**	**0.0174**	**-0.65**	**0.0005**
Spirochaetes	-0.39	0.0569	**-0.61**	**0.0011**	**-0.58**	**0.0024**	-0.16	0.4534	-0.04	0.8574	-0.38	0.0613
Synergistetes	-0.17	0.4062	**-0.45**	**0.0249**	-0.39	0.0571	-0.13	0.539	-0.09	0.6829	-0.30	0.1512
Tenericutes	**-0.60**	**0.0016**	**-0.71**	**< 0.0001**	**-0.64**	**0.0006**	-0.39	0.0552	-0.17	0.4034	**-0.55**	**0.0040**

^1^Bold correlation coefficients (r) with *p* < 0.05, and underlined correlation coefficients (r) with r > 0.5 (strong correlation)

**Table 5:** Correlation coefficients (r) between oral health scores, plaque scores, calculus scores, gingivitis scores, pocket scores, pH, and bacterial phyla in supragingival samples (*n* = 26)^1^

Phyla	Salivary pH		Pocket Score		Gingivitis Score		Calculus Score		Plaque Score		Oral Health Score	
	r	p	r	p	r	p	r	p	r	p	r	p
Actinobacteria	**0.52**	**0.007**	**0.53**	**0.0059**	**0.47**	**0.0143**	0.37	0.0654	0.20	0.3319	**0.46**	**0.0188**
Bacteroidetes	**-0.70**	**< 0.0001**	**-0.83**	**< 0.0001**	**-0.74**	**< 0.0001**	**-0.62**	**0.0008**	**-0.40**	**0.0411**	**-0.76**	**< 0.0001**
Firmicutes	**-0.43**	**0.0296**	**-0.53**	**0.0051**	-0.38	0.0567	**-0.41**	**0.0365**	-0.37	0.0596	**-0.52**	**0.0066**
Proteobacteria	**0.64**	**0.0004**	**0.76**	**< 0.0001**	**0.64**	**0.0004**	**0.57**	**0.0025**	**0.46**	**0.0187**	**0.72**	**< 0.0001**
SR1	**-0.51**	**0.008**	**-0.60**	**0.0012**	**-0.63**	**0.0005**	**-0.43**	**0.0265**	**-0.46**	**0.0178**	**-0.60**	**0.0011**
Tenericutes	**-0.64**	**0.0005**	**-0.60**	**0.0012**	**-0.56**	**0.0029**	-0.37	0.0654	-0.21	0.2933	**-0.50**	**0.0092**

^1^Bold correlation coefficients (r) with *p* < 0.05, and underlined correlation coefficients (r) with r > 0.5 (strong correlation)

**Table 6:** Correlation coefficients (r) between oral health scores, plaque scores, calculus scores, gingivitis scores, pocket scores, pH, and bacterial phyla in saliva samples (*n* = 26)^1^

Phyla	Salivary pH		Pocket Score		Gingivitis Score		Calculus Score		Plaque Score		Oral Health Score	
	r	p	r	p	r	p	r	p	r	p	r	p
Euryarchaeota	**0.42**	**0.0349**	**0.42**	**0.0321**	**0.45**	**0.0206**	0.32	0.1168	0.27	0.1769	**0.42**	**0.035**
Bacteroidetes	**-0.47**	**0.0156**	-0.35	0.0799	-0.31	0.1265	-0.11	0.6035	0.11	0.5966	-0.18	0.3751
Chloroflexi	**0.46**	**0.0175**	**0.58**	**0.0017**	**0.58**	**0.0017**	**0.50**	**0.0089**	**0.41**	**0.0377**	**0.61**	**0.0010**
Firmicutes	-0.33	0.0971	**-0.47**	**0.0163**	**-0.44**	**0.0252**	-0.13	0.5307	-0.20	0.3275	-0.34	0.0924
Synergistetes	**0.60**	**0.0013**	**0.73**	**< 0.0001**	**0.67**	**0.0002**	**0.51**	**0.0076**	**0.46**	**0.018**	**0.69**	**< 0.0001**
Tenericutes	**-0.42**	**0.0315**	-0.36	0.0681	-0.30	0.143	-0.38	0.0522	-0.27	0.183	**-0.40**	**0.0413**

^1^Bold correlation coefficients (r) with *p* < 0.05, and underlined correlation coefficients (r) with -r > 0.5 (strong correlation)

**Table 7:** Correlation coefficients (r) between oral health scores, plaque scores, calculus scores, gingivitis scores, pocket scores, pH and bacteria genera in subgingival samples^1^

Genera	Salivary pH		Pocket Score		Gingivitis Score		Calculus Score		Plaque Score		Oral Health Score	
	r	p	r	p	r	p	r	p	r	p	r	p
*Actinomyces*	**0.44**	**0.0294**	**0.68**	**0.0002**	**0.61**	**0.0011**	0.29	0.1531	0.31	0.1365	**0.54**	**0.0050**
*Corynebacterium*	**0.62**	**0.0009**	**0.78**	**< 0.0001**	**0.70**	**< 0.0001**	**0.40**	**0.0480**	0.32	0.1235	**0.63**	**0.0007**
*Euzebya*	**0.41**	**0.0429**	**0.63**	**0.0008**	**0.49**	**0.0134**	0.16	0.4307	0.22	0.3003	**0.43**	**0.0318**
*Bacteroides*	**-0.44**	**0.0268**	-0.35	0.0894	-0.31	0.1292	-0.23	0.2583	0.00	0.9988	-0.26	0.2010
*Parabacteroides*	-0.14	0.5175	-0.36	0.0753	**-0.41**	**0.0413**	-0.03	0.8750	-0.11	0.6167	-0.23	0.2653
*Porphyromonas*	-0.64	0.0006	**-0.78**	**< 0.0001**	**-0.74**	**< 0.0001**	**-0.53**	**0.0070**	**-0.44**	**0.0289**	**-0.72**	**< 0.0001**
*Tannerella*	-0.15	0.4702	**-0.40**	**0.0492**	-0.21	0.3027	0.01	0.9545	-0.20	0.3456	-0.23	0.2610
*Prevotella*	**-0.48**	**0.0152**	**-0.62**	**0.0009**	**-0.62**	**0.0009**	-0.35	0.0856	-0.16	0.4521	**-0.50**	**0.0113**
*Capnocytophaga*	**0.49**	**0.0133**	**0.63**	**0.0007**	**0.58**	**0.0022**	0.23	0.2601	0.13	0.5482	**0.44**	**0.0263**
*Peptococcus*	**-0.53**	**0.0065**	**-0.59**	**0.0018**	**-0.64**	**0.0006**	**-0.45**	**0.0250**	-0.27	0.1946	**-0.56**	**0.0037**
*Filifactor*	**-0.53**	**0.0069**	**-0.70**	**0.0001**	**-0.68**	**0.0002**	**-0.45**	**0.0245**	-0.34	0.096	**-0.63**	**0.0008**
*Proteocatella*	**0.58**	**0.0024**	**0.61**	**0.0011**	**0.61**	**0.0012**	0.26	0.2046	0.13	0.5251	**0.45**	**0.0234**
*Schwartzia*	-0.08	0.6931	-0.36	0.0771	**-0.42**	**0.0366**	-0.17	0.4195	-0.14	0.5122	-0.29	0.1529
*Fusibacter*	**-0.45**	**0.0226**	**-0.78**	**< 0.0001**	**-0.73**	**< 0.0001**	**-0.49**	**0.0133**	**-0.58**	**0.0024**	**-0.75**	**< 0.0001**
*Helcococcus*	**-0.48**	**0.0153**	-0.30	0.1419	-0.19	0.3632	-0.32	0.1207	-0.19	0.3593	-0.32	0.1165
*p-75-a5*	**0.44**	**0.0272**	0.39	0.0548	**0.47**	**0.0174**	0.23	0.2612	-0.05	0.7997	0.28	0.1713
*Fusobacterium*	0.20	0.3373	0.39	0.0531	**0.54**	**0.0054**	0.22	0.2831	0.14	0.5161	0.34	0.0955
*Leptotrichia*	**0.53**	**0.0062**	**0.71**	**< 0.0001**	**0.69**	**0.0001**	0.26	0.2115	0.20	0.3459	**0.52**	**0.0080**
*Lautropia*	0.37	0.0657	**0.46**	**0.0210**	**0.50**	**0.0106**	0.27	0.1991	0.07	0.7411	0.36	0.0757
*Lampropedia*	0.40	0.0448	**0.49**	**0.0132**	**0.53**	**0.0060**	0.49	0.0131	0.21	0.3088	**0.51**	**0.0099**
*Neisseria*	0.32	0.1198	**0.46**	**0.0201**	0.33	0.1116	0.07	0.7357	0.26	0.2025	0.32	0.1149
*Propionivibrio*	0.23	0.2782	0.33	0.1033	**0.51**	**0.0085**	0.38	0.0617	0.19	0.3560	0.39	0.0522
*Desulfobulbus*	0.47	0.0166	**0.48**	**0.0147**	**0.53**	**0.0063**	0.33	0.1051	0.15	0.4772	**0.42**	**0.0352**
*Desulfomicrobium*	0.17	0.4212	0.30	0.1436	**0.48**	**0.0164**	**0.50**	**0.0105**	0.33	0.1103	**0.46**	**0.0199**
*Arcobacter*	**0.43**	**0.0311**	0.30	0.1470	0.23	0.2630	0.24	0.2575	0.30	0.1401	0.32	0.1139
*Pasteurella*	0.32	0.1233	**0.57**	**0.0028**	**0.46**	**0.0206**	0.26	0.2079	0.31	0.1302	**0.47**	**0.0178**
*Moraxella*	**0.49**	**0.0126**	**0.68**	**0.0002**	**0.52**	**0.0072**	0.29	0.1623	0.21	0.3152	**0.50**	**0.0108**
*Treponema*	-0.38	0.0595	**-0.61**	**0.0012**	**-0.58**	**0.0026**	-0.15	0.4738	-0.04	0.8449	-0.38	0.0635
*TG5*	-0.18	0.3845	**-0.46**	**0.0214**	**-0.40**	**0.0488**	-0.13	0.5280	-0.09	0.6764	-0.30	0.1409
*Acholeplasma*	**-0.66**	**0.0003**	**-0.69**	**0.0001**	**-0.61**	**0.0014**	**-0.46**	**0.0199**	-0.33	0.1120	**-0.62**	**0.0011**

^1^Bold correlation coefficients (r) with *p* < 0.05, and underlined correlation coefficients (r) with r > 0.5 (strong correlation)

**Table 8:** Correlation coefficients (r) between oral health scores, plaque scores, calculus scores, gingivitis scores, pocket scores, pH and bacteria genera in supragingival samples^1^

Genera	Salivary pH		Pocket Score		Gingivitis Score		Calculus Score		Plaque Score		Oral Health Score	
	r	p	r	p	r	p	r	p	r	p	r	p
*Actinomyces*	0.26	0.2004	**0.57**	**0.0024**	**0.50**	**0.0087**	0.19	0.3400	0.20	0.3302	**0.41**	**0.0376**
*Corynebacterium*	**0.54**	**0.0047**	**0.52**	**0.0069**	**0.46**	**0.0191**	0.32	0.1099	0.23	0.2614	**0.44**	**0.0235**
*Leucobacter*	**-0.41**	**0.0372**	**-0.50**	**0.0094**	**-0.57**	**0.0024**	-0.04	0.8286	-0.12	0.5685	-0.31	0.1278
*Paludibacter*	**0.57**	**0.0023**	**0.59**	**0.0015**	**0.52**	**0.0066**	0.30	0.1309	0.09	0.6701	**0.43**	**0.0276**
*Porphyromonas*	**-0.70**	**< 0.0001**	**-0.75**	**< 0.0001**	**-0.60**	**0.0013**	**-0.47**	**0.0164**	-0.26	0.1950	**-0.62**	**0.0008**
*Prevotella*	-0.13	0.5346	-0.30	0.1377	-0.28	0.1699	**-0.41**	**0.0353**	**-0.47**	**0.0146**	**-0.45**	**0.0227**
*Capnocytophaga*	**0.44**	**0.0240**	**0.45**	**0.0213**	0.27	0.1886	0.17	0.4067	0.16	0.4378	0.32	0.1144
*Clostridium*	0.29	0.1473	**0.42**	**0.0329**	**0.50**	**0.0086**	0.28	0.1671	0.18	0.3766	0.38	0.0561
*Catonella*	0.02	0.9084	-0.15	0.4692	-0.14	0.4805	**-0.40**	**0.0427**	-0.38	0.0533	-0.34	0.0883
*Peptococcus*	-0.34	0.0879	**-0.40**	**0.0414**	-0.29	0.1519	-0.34	0.0877	-0.37	0.0613	**-0.43**	**0.0285**
*Filifactor*	**-0.65**	**0.0003**	**-0.69**	**0.0001**	**-0.57**	**0.0026**	-0.31	0.1235	-0.16	0.4234	**-0.50**	**0.0096**
*Proteocatella*	**0.42**	**0.0316**	0.39	0.0505	**0.45**	**0.0199**	0.23	0.2664	-0.02	0.9324	0.28	0.1589
*Fusibacter*	**-0.52**	**0.0066**	**-0.59**	**0.0016**	**-0.48**	**0.0130**	-0.16	0.4217	0.00	0.9927	-0.35	0.0837
*Helcococcus*	**-0.55**	**0.0034**	**-0.55**	**0.0038**	**-0.51**	**0.0083**	**-0.47**	**0.0148**	-0.34	0.0941	**-0.55**	**0.0036**
*p-75-a5*	-0.04	0.8427	-0.14	0.4835	-0.14	0.5106	**-0.42**	**0.0309**	-0.36	0.0686	-0.34	0.0877
*Leptotrichia*	**0.42**	**0.0341**	**0.59**	**0.0016**	**0.44**	**0.0239**	0.30	0.1368	0.17	0.3946	**0.45**	**0.0224**
*Lampropedia*	0.27	0.1818	**0.42**	**0.0307**	0.30	0.1308	**0.47**	**0.0157**	**0.54**	**0.0044**	**0.54**	**0.0046**
*Neisseria*	**0.49**	**0.0102**	**0.57**	**0.0023**	**0.40**	**0.0418**	0.32	0.1100	0.32	0.1151	**0.48**	**0.0123**
*Desulfobulbus*	0.38	0.0586	**0.57**	**0.0025**	**0.64**	**0.0004**	**0.49**	**0.0107**	0.35	0.0761	**0.59**	**0.0017**
*Campylobacter*	**-0.51**	**0.0083**	**-0.67**	**0.0002**	**-0.56**	**0.0030**	**-0.52**	**0.0061**	**-0.53**	**0.0050**	**-0.68**	**0.0001**
*Acholeplasma*	**-0.57**	**0.0026**	**-0.55**	**0.0033**	**-0.51**	**0.0083**	**-0.45**	**0.0205**	-0.19	0.3562	**-0.50**	**0.0087**

^1^Bold correlation coefficients (r) with *p* < 0.05, and underlined correlation coefficients (r) with r > 0.5 (strong correlation)

**Table 9:** Correlation coefficients (r) between oral health scores, plaque scores, calculus scores, gingivitis scores, pocket scores, pH and bacteria genera in saliva samples^1^

Genera	Salivary pH		Pocket Score		Gingivitis Score		Calculus Score		Plaque Score		Oral Health Score	
	r	p	r	p	r	p	r	p	r	p	r	p
*Paludibacter*	0.32	0.1118	**0.45**	**0.0205**	**0.46**	**0.0183**	0.15	0.4569	0.10	0.6351	0.31	0.1186
*Parabacteroides*	**0.47**	**0.0156**	0.36	0.0748	0.26	0.2067	0.35	0.0763	0.36	0.074	**0.41**	**0.0386**
*Prevotella*	**-0.42**	**0.0331**	-0.31	0.1288	-0.23	0.2603	-0.35	0.0767	-0.03	0.8993	-0.29	0.1478
*SHD-231*	**0.46**	**0.0175**	**0.58**	**0.0017**	**0.58**	**0.0017**	**0.50**	**0.0089**	**0.41**	**0.0377**	**0.61**	**0.0010**
*Clostridium*	**0.39**	**0.0499**	**0.50**	**0.0095**	**0.52**	**0.0066**	**0.48**	**0.0134**	0.33	0.0988	**0.53**	**0.0050**
*Catonella*	0.28	0.1699	-0.05	0.8036	-0.18	0.3918	-0.33	0.099	**-0.41**	**0.0390**	-0.28	0.1589
*Oscillospira*	**0.50**	**0.0089**	**0.58**	**0.0021**	**0.61**	**0.0009**	**0.45**	**0.0203**	0.35	0.0795	**0.57**	**0.0024**
*Schwartzia*	**0.48**	**0.0126**	**0.50**	**0.0101**	**0.40**	**0.0401**	0.24	0.2321	0.17	0.4046	0.38	0.0541
*Fusibacter*	0.32	0.1076	0.37	0.0664	0.38	0.0579	**0.39**	**0.0468**	0.34	0.0943	**0.43**	**0.0267**
*Anaerovorax*	**0.51**	**0.0083**	**0.49**	**0.0119**	**0.50**	**0.0091**	0.22	0.2903	0.05	0.8204	0.34	0.0869
*Fusobacterium*	-0.21	0.3012	**-0.40**	**0.0415**	**-0.40**	**0.0444**	-0.25	0.2237	-0.27	0.1854	-0.37	0.0601
*Propionivibrio*	-0.01	0.9801	0.19	0.3432	0.29	0.1577	0.35	0.078	**0.43**	**0.0291**	0.37	0.0656
*Desulfobulbus*	0.29	0.1461	**0.41**	0.0355	**0.41**	**0.0387**	0.33	0.0974	0.35	0.0840	**0.43**	**0.0265**
*Desulfomicrobium*	**0.55**	**0.0036**	**0.71**	**< 0.0001**	**0.63**	**0.0005**	**0.57**	**0.0022**	**0.47**	**0.0166**	**0.71**	**< 0.0001**
*Desulfovibrio*	**0.46**	**0.0171**	**0.50**	**0.0087**	**0.48**	**0.0134**	**0.47**	**0.0147**	0.32	0.1064	**0.53**	**0.0056**
*Pasteurella*	-0.38	0.0587	-0.26	0.1933	-0.31	0.1215	-0.39	0.0515	**-0.42**	**0.0317**	**-0.41**	**0.0383**
*Enhydrobacter*	**0.45**	**0.0206**	**0.43**	**0.0285**	**0.43**	**0.0289**	0.26	0.1988	0.23	0.2497	0.38	0.0533
*TG5*	**0.61**	**0.0009**	**0.75**	**< 0.0001**	**0.68**	**0.0001**	**0.51**	**0.0076**	**0.46**	**0.0176**	**0.70**	**< 0.0001**
*Acholeplasma*	**-0.42**	**0.0321**	-0.36	0.0736	-0.28	0.1679	-0.33	0.0988	-0.25	0.2184	-0.37	0.0615

^1^Bold correlation coefficients (r) with *p* < 0.05, and underlined correlation coefficients (r) with r > 0.5 (strong correlation)

Relative abundance of *Actinomyces* was positively correlated with OHS, gingivitis and pocket score, and salivary pH in SUB samples, and with the same oral scores but not with salivary pH in SUP samples. Relative abundance of *Corynebacterium* was positively correlated with all oral scores (minus plaque score) and salivary pH in SUB samples, and with the same oral scores as in SUB samples (minus calculus score) and salivary pH in SUP samples. Relative abundance of *Porphyromonas* was negatively correlated with all oral scores and salivary pH in SUB samples, and with same scores (minus plaque score) and salivary pH in SUP samples. Relative abundance of *Prevotella* was negatively correlated with OHS, gingivitis and pocket score, and salivary pH in SUB samples, and with OHS, plaque and calculus score in SUP samples, and only with salivary pH in SAL samples. Relative abundance of *Capnocytophaga* was positively correlated with OHS, gingivitis and pocket score, and salivary pH in SUB samples, and with pocket score and salivary pH in SUP samples. Relative abundance of *Peptococcus* was negatively correlated with all oral scores (minus plaque score) and salivary pH in SUB samples, and with OHS and pocket score in SUP samples. Relative abundance of *Filifactor* was negatively correlated with all oral scores (minus plaque score) and salivary pH in SUB samples, and with the same oral scores as in SUB samples (minus calculus score) and salivary pH in SUP samples. Relative abundance of *Helcococcus* was negatively correlated with all oral scores (minus plaque score) and salivary pH in SUP samples, and only with salivary pH in SUB samples. Relative abundance of *Leptotrichia* was positively correlated with OHS, gingivitis and pocket score, and salivary pH in plaque samples. Relative abundance of *Lampropedia* was positively correlated with OHS, gingivitis and pocket score, and salivary pH in SUB samples, and with OHS, plaque, calculus, and pocket score in SUP samples. Relative abundance of *Neisseria* was positively correlated with OHS, gingivitis and pocket score, and salivary pH in SUP samples, and with pocket score in SUB samples. Relative abundance of *Propionivibrio* was positively correlated with gingivitis score in SUB samples, and with plaque score in SAL samples. Relative abundance of *Desulfobulbus* was positively correlated with all oral scores (minus plaque score) in SUP samples, and with the same score as in SUP samples (minus calculus score) in SAL and SUB samples, plus salivary pH in SUB samples. Relative abundance of *Desulfomicrobium* was positively correlated with all oral scores and salivary pH in SAL samples, and with OHS, calculus and gingivitis score in SUB samples. Relative abundance of *Acholeplasma* was negatively correlated with all oral scores (minus plaque score) and salivary pH in plaque samples, and with salivary pH in SAL samples.

## Discussion

The oral microbiota of dogs in the present study was highly rich and diverse, consistent with previous studies [9–12, 24, 25]. However, it has previously been described that the Shannon diversity index was significantly larger for SUP and significantly smaller for SAL samples compared to all other niches [18]. In contrast to earlier findings in dogs, however, in the present study, SAL and SUB had similar Shannon index values that were higher than that of SUP. The lower diversity index value observed in the previous study for the canine saliva population may be due to the fact that the saliva was stimulated before collection, thus diluting the salivary microbiota. In the present study, the saliva was not stimulated, and therefore the samples in the present study were not diluted. Similar to the present study, in humans, studies focusing on multiple oral habitats described diversity parameters to be highest for both SUP and SAL samples [16, 26–28].

Davis et al. [9] reported that SUB samples from dogs of varying oral health statuses (72 with healthy gingiva; 77 with gingivitis; 74 with mild periodontitis) were colonized largely by Firmicutes, Bacteroidetes, Proteobacteria, Actinobacteria, Fusobacteria, and Spirochaetes regardless of disease stage. Additionally, in the healthy cohort, Proteobacteria and Bacteroidetes were the most abundant phyla; *Porphyromonas*, *Moraxella*, and *Bergeyella* were the most abundant genera in all dogs, and particularly higher in healthy animals [9]. In another study evaluating a composite oral sample of healthy dogs (*n* = 6), whereby samples were collected by brushing the gums, tongue, teeth, and cheeks, the phyla Bacteroidetes, Proteobacteria, and Firmicutes predominated; the most commonly identified genera were *Porphyromonas*, *Fusobacterium*, *Capnocytophaga*, *Derxia*, and *Moraxella* [25]. Additionally, in a healthy cohort (14 dogs), SUP samples were collected using plastic microbiological loops, buccal and tongue dorsum mucosa were collected using a CytoSoft cytology brush, and stimulated whole mouth saliva was collected using cotton wool swabs. In that study, Proteobacteria, Firmicutes, and Bacteroidetes were the most abundant phyla across all niches, although the ranking of these varied among niche [18]. Similarly, in the present study, Bacteroidetes, Proteobacteria, and Firmicutes were the predominant phyla in the SUB and SAL samples. *Porphyromonas* was the most abundant genus, followed by *Fusobacterium*, *Treponema*, *Enhydrobacter*, and *Moraxella*. However, *Capnocytophaga* and *Bergeyella* had a low abundance. In SUP samples, Proteobacteria, Bacteroidetes, and Firmicutes were the predominant phyla, with *Porphyromonas*, *Moraxella*, *Enhydrobacter*, *Fusobacterium*, *Corynebacterium*, and *Actinomyces* being the predominant genera. The high variability of the oral microbiota among studies may be due to differences between animals, facilities (water, food, products used for cleaning), dental prophylaxis, type of swabs, extraction protocols, and/or the amplified 16S rRNA gene hypervariable region used in the microbiota analysis. Differences may also be due to interactions between the saliva, nutrient sources, host cell type, immunological factors, and exogenous factors such as oxygen availability and oral intake [9, 16].

In the present study, the microbiota populations were quite different among oral habitats (SAL, SUB, and SUP). The variation between SUP and SAL is consistent with data from a previous canine study whereby microbiota of SUP and oral swabs were shown to be distinct [24]. These data are also similar to data from a human study that demonstrated that the buccal mucosa, gingivae, and hard palate microbiota populations were similar to one another and different than the populations present in saliva, tongue, tonsils and throat, and SUP and SUB that were similar to one another [16]. The distinct microbiota communities of these microenvironments within the oral cavity are likely due to the differences in oxygen tension, pH, and mucosal surface characteristics [29, 30].

In a previous study of 30 healthy adult Beagle dogs, SUP plaque and swabs from gums, tongue, and cheeks were sampled, reporting that Firmicutes and Spirochaetes were predominant in the plaque environment, and Proteobacteria and Firmicutes were predominant in the oral swabs [24]. Similar data were reported in the present study, with the microbiota of SAL having lower Firmicutes than the tooth plaque sites. In contrast to the previous dog study [24] where the relative abundance of Actinobacteria was higher in SUP compared to an oral swab, the relative abundance of Actinobacteria was higher in SAL compared to tooth plaque site samples (SUB and SUP) in the present study. In a previous dog study, *Treponema* relative abundance was greater in SUP samples than oral swab samples [composite oral swabs - flocked nylon-tipped BD Liquid Amies Elution swabs (Becton, Dickinson and Company, USA) - collected by swabbing the gums, tongue, and cheeks for 10–15 s], *Actinomyces* and *Pasteurella* relative abundances were greater in swab samples than SUP samples, and there was no difference in *Porphyromonas* relative abundance among habitats [24]. Contrary, in the present study, relative abundance *of Actinomyces* was higher in the SUP than SAL samples, relative abundance *of Treponema* and *Porphyromonas* were lower in the SUP than SAL samples, and relative abundance *of Pasteurella* was similar between SUP and SAL samples.

Furthermore, in a dog study whereby a checkerboard DNA–DNA hybridization of human probes was used, five intra-oral habitats (SUB, SUP, the tongue, tonsils and cheek mucosa) were evaluated in seven Beagle dogs [17]. In that study, the prevalence of 26 species were different between SUP and SUB plaque samples, with 20 of them being higher in SUB plaque [17]. SUP plaque contained higher proportions of *P. gingivalis*, *F. periodonticum*, *F. nucleatum* ss. vincentii, *A. actinomycetemcomitans*, *Prevotella acnes* and *A. naeslundii* genospecies 2-like species [17]. In the present study, SUP contained higher proportions of *Actinomyces*, *Corynebacterium*, *Leucobacter, Capnocytophaga*, *Bergeyella*, *Oscillospira*, *p-75-a5*, *Lautropia*, *Lampropedia*, *Desulfobulbus*, *Arcobacter*, *Pasteurella*, *Enhydrobacter*, and *Moraxella*. In the previous dog study, the microbial profiles of the soft habitats (i.e., cheek and tongue mucosa, tonsils) and tooth plaque sites were markedly different, with 19 of 40 species differing among sample locations [17]. *P. gingivalis*, *T. denticola*, *Tannerella forsythia*, *S. constellatus*, *C. rectus* and *C. showae*-like species were present in higher proportions on tooth plaque habitats [17]. In the present study, the relative abundances of 7 genera were different among saliva and tooth plaque habitats. *Tannerella*, *Peptostreptococcus*, *Schwartzia*, and *Neisseria* were present in higher proportions on tooth plaque sites.

Similar to the previous study, *Tannerella* was present in a higher proportion on tooth plaque sites. One of the reasons could be that *Tannerella* are capable of producing proteolytic enzymes that can degrade host periodontal tissues and compromise the host immune system. *Tannerella* also possesses a surface-associated putative adhesin that serves as ligands to other bacteria (*Fusobacterium*), which provide this bacterial group with the ability to facilitate the development of complex communities and plaque formation [31–40]. *Peptostreptococcus* are capable of inducing a potent inflammatory reaction in macrophages, producing proteases that permit it to penetrate to the basement membrane, and creating a carbohydrate-mediated coaggregation with *Fusobacterium* and *Porphyromonas* [41–43], which also enable this bacterial genera to facilitate plaque development. In cats, *Schwartzia* was reported to be associated with gingivitis [44]. In humans, periodontal patients not only had higher relative abundances of periopathogens, but also of other taxa (*Anaeroglobus*, *Bulleidia*, *Desulfobulbus*, *Filifactor*, *Mogibacterium*, *Phocaeicola*, *Schwartzia*, or *TM7*) whose role in oral health are not well-established but may be targeted in future research [45]. In dogs, the primary colonizers of the tooth surface appear to be *Neisseria* and *Moraxella* [10, 46, 47]. Therefore, it was expected that a higher proportion of these bacterial groups would be measured in plaque habitats.

In a human study, it was suggested that various oral habitats (buccal mucosa, keratinized gingiva, hard palate, throat, palatine tonsils, tongue dorsum, SAL, SUP, SUB) could be characterized and then easily sampled sites (e.g., SAL, tongue) could be used as surrogate markers for the others [16]. Similarly in animals, SAL and SUP samples are relatively easy to collect and do not require sedation for the majority of the animals. The data from the present study and that of a previous study [24], however, shows that the use of the oral salivary swabs to assess the oral plaque microbiota is not recommended because their communities are distinct from those of the plaque populations and would most likely be misleading. In a previous study, higher relative abundance of *Treponema* and Clostridiales in plaque, and higher relative abundance of *Psychrobacter*, *Mannheimia*, and *Pasteurella* in swab samples (gums, tongue, and cheeks), demonstrated that plaque microbiota harbor greater populations of anaerobic and biofilm-associated taxa [24]. Similarly in the present study, *Paludibacter*, *Filifactor, Peptostreptococcus, Fusibacter, Anaerovorax, Fusobacterium, Leptotrichia, Desulfomicrobium*, and *TG5* (anaerobic bacteria) were enriched in SUB samples. *Actinomyces*, *Corynebacterium*, *Leucobacter*, *Euzebya*, *Capnocytophaga*, *Bergeyella*, *Lautropia, Lampropedia, Desulfobulbus, Enhydrobacter*, and *Moraxella* (aerobic and anaerobic bacteria) were enriched in SUP samples. *Prevotella, SHD-231, Helcococcus, Treponema*, and *Acholeplasma* (aerobic and anaerobic bacteria) were enriched in SAL samples.

Identifying the relationships between oral microbiota and periodontal disease is extremely important to understand the disease process and how to prevent or treat it. Past studies have focused on *Porphyromonas*, as it is well known to be one of the most important bacteria for the development and progression of periodontal disease in humans [48–51]. In past studies with dogs, *Porphyromonas* was the most abundant genus, being particularly higher in healthy dogs [9, 25]. In the present study, *Porphyromonas* was highly prevalent, providing strong evidence that they are part of the commensal oral microbiome. The data from the current and past studies suggest that instead of having a complete absence of pathogenic organisms in the normal microbiota, disease occurs when there is an imbalance [52, 53]. Nevertheless, other groups of bacteria seem to be key components of periodontal disease in dogs, including *Peptostreptococcus*, *Actinomyces*, and Peptostreptococcaceae that have been shown to be the most predominant taxa in dogs with mild periodontitis, with *Corynebacterium canis* being more abundant in dogs with mild periodontitis and gingivitis, and *Leptotrichia* sp., *Neisseria canis*, and an uncultured *Capnocytophaga* sp. being associated with gingivitis [9].

In the present study we correlated bacterial genera and oral scores, and in SUB samples, *Actinomyces*, *Corynebacterium*, and *Leptotrichia* were strongly and positively correlated with higher pocket score, gingivitis score, and OHS. We also observed a strong positive correlation between *Capnocytophaga* and pocket and gingivitis scores. In SUP samples, a strong positive correlation between *Actinomyces* and pocket and gingivitis scores, and strong positive correlations between *Corynebacterium*, *Leptotrichia*, and *Neisseria* and pocket score were observed. *Actinomyces* belong to the group of bacteria that can overcome the immune barrier, pass through endothelial gaps and pores, penetrate the bloodstream. Therefore, it plays a significant role in gingivitis and the progression of periodontal diseases because they are able to cause inflammation, periapical lesion, and induce soft and hard tissue destruction [54–59]. *Capnocytophaga* spp. possess a trypsin-like enzyme and are considered to be periodontopathic [60]. *Leptotrichia* species typically colonize the oral cavity and have been reported to participate in oral disease in humans (gingivitis, necrotizing ulcerative gingivitis, adult/juvenile periodontitis, ‘‘refractory’’ periodontitis). *L. buccalis* is highly saccharolytic and produces lactic acid, a property that may implicate participation in tooth damage [61–66]. Additionally, non-plaque induced gingival lesions can result from specific bacterial pathogens such as *Neisseria gonorrhea* [67]. Therefore the correlation of those bacterial genera with higher gingivitis and pocket scores was expected. Even though ease of access and the lack of anesthesia would reduce the cost and complication of saliva collection, bacteria related to the development of periodontal disease and gingivitis are present in greater concentrations in oral plaque. Therefore, plaque collection is suggested. Unreliable data coming from SAL samples would likely lead to inaccurate diagnosis and monitoring of oral health, potentially delaying proper care and tooth cleaning that would worsen periodontal disease.

Limitations of the present study are that the (1) animals with severe oral disease were not included; this information would help understand differences in the microbiota community due to periodontal disease and (2) samples were only taken from one point of collection, with longitudinal samples over time providing a better description of the changes in oral microbiota during the development of oral disease.

## Conclusions

The present study provided a broad characterization of the oral microbiome of healthy dogs. Because oral health scores differed across the population, the data may serve as a foundation for the study of healthy and diseased dogs in the future. Our results demonstrate the differences that exist among the salivary, subgingival plaque, and supragingival plaque samples of dogs. Salivary samples do not require sedation and are easy to collect, but do not accurately represent the populations most important to oral disease. *Actinomyces, Corynebacterium, Capnocytophaga*, *Leptotrichia*, and *Neisseria* were associated with higher oral health scores (worsened health) in plaque samples, which might be useful for future studies to understand the bacterial groups that are responsible for the development and progression of periodontal disease. A natural progression of this work is to analyze samples from progressive stages of periodontal disease, to validate the use of microbiota markers for disease.

## Methods

### Animals

 Twenty-six adult female Beagle dogs (4.0 ± 1.2 year old) were used for saliva and plaque collection. Collection was done over the course of a few days, but methods were exactly the same for all. Dogs were housed individually in pens (1.0 m wide by 1.8 m long) in a humidity- and temperature-controlled animal facility. All dogs had free access to water and were fed a commercial dry kibble diet for several mo prior to sample collection and scoring. None of the dogs received antibiotics or probiotics for several mo before scoring and plaque sample collection. All procedures were approved by the University of Illinois Institutional Animal Care and Use Committee prior to experimentation.

### Anesthesia methods

All dogs had their food withheld for at least 12 h prior to anesthesia, but were allowed water until 30 min prior to sedation. Dogs were premedicated with butorphanol (0.3 mg/kg). Twenty to 30 min after pre-medication, the fur over the cephalic vein was clipped, the site was aseptically prepared, and a 20-gauge intravenous catheter was placed in the cephalic vein for administration of anesthetic agents and intravenous fluids. Dogs were pre-oxygenated and anesthesia was induced with etomidate following either midazolam (0.3 mg kg^− 1^), lidocaine (2 mg kg^− 1^), or physiologic saline (1 mL) administered intravenously. Heart rate, invasive arterial blood pressure, respiratory rate, and intraocular pressure were recorded following butorphanol sedation, after co-induction administration, after etomidate administration, and following intubation. Dogs were orotracheally intubated and transferred to isoflurane to maintain anesthesia. Intravenous fluids were run at 5 mL/kg/hr throughout anesthesia and active heating with a forced air warmer was provided to maintain normothermia. Cardiovascular and respiratory function was monitored continuously using an anesthetic multiparameter monitor (electrocardiogram, oscillometric blood pressure, capnograph, pulse oximeter, and temperature). Supplementary anesthetic agents and cardiovascular support were administered as needed based on the decision of the attending anesthesiologist.

### Salivary pH

Salivary pH was measured using pH strips (Fisherbrand™ Plastic pH Strips; pH range 0–14) on the same day and time of dental scoring. All dogs had their food withheld for at least 12 h prior salivary pH measurements, using two strips on each side per dog (4 total). The salivary pH reported was the mean of the 4 strips. Saliva samples were collected where it naturally pools (in the cheek pouch and under the tongue) for 30 s.

### Dental scoring

Gingivitis, plaque, and calculus scoring were conducted by a board-certified veterinary dentist according to a modified version of previous scoring systems [68, 69]. For each measurement, the 4th premolar and 1st molar teeth on the upper (maxilla) and lower (mandible) jaw were scored. These two teeth were chosen because they are the teeth from which plaque samples for microbiota analysis were collected. Thus the dental score data would be more compatible with the sample collection site.

To assess gingivitis, a periodontal probe was placed subgingivally on the buccal side of each tooth and values were assigned via visual assessment of inflammation and bleeding upon probing (0 = normal gingiva: no inflammation; 1 = very mild gingivitis: slight change in color, slight edema and no bleeding on probing; 2 = mild gingivitis: redness, edema, glazing of surface, bleeding on probing within 30-seconds; 3 = moderate gingivitis: redness, edema, immediate bleeding on probing; 4 = severe gingivitis: ulceration, spontaneous bleeding and profuse bleeding on probing). Each tooth was graded by the average of the three scores obtained per tooth. The score for each dog was the mean score for all teeth scored.

Plaque levels were evaluated using Trace Disclosing Solution (Young Dental, Earth City, MO, USA) to cover the teeth followed by a gentle rinse of water to remove the excess. The gingival and occlusal half of each tooth was scored for coverage (0 = no detectable plaque; 1 = scattered plaque covering less than 24 % of the buccal tooth surface; 2 = plaque covering between 25 and 49 % of the buccal tooth surface; 3 = plaque covering between 50 and 74 % of the buccal tooth surface; plaque covering more than 75 % of the buccal tooth surface) and thickness (1 = light; 2 = moderate; 3 = heavy). The gingival and occlusal values for each tooth were averaged to obtain a tooth total score. The average plaque coverage was multiplied by the average of plaque thickness to obtain a whole mouth mean calculus score for each animal.

The disclosed plaque was removed by gentle tooth brushing and rinsing with a dental air-water syringe. The tooth was then air-dried. Calculus scores were based on visual assessment of coverage (0 = no detectable calculus; 1 = scattered calculus covering less than 24 % of the buccal tooth surface; 2 = calculus covering between 25 and 49 % of the buccal tooth surface; 3 = calculus covering between 50 and 74 % of the buccal tooth surface; 4 = calculus covering more than 75 % of the buccal tooth surface) and thickness (< 0.5 mm = 1; 0 0.5 − 1.0 mm = 2; >1.0 mm = 3) on the mesial, buccal, and distal portions of the tooth. The tooth score is the average of the scores for each of the three tooth surfaces. The average of calculus coverage was multiplied by the average of calculus thickness to obtain a whole mouth mean calculus score for each animal.

Pocket depth was based on height from bottom of pocket to gingival margin, <2mm = normal sulcus; >2 and < 3mm = slight; >3 and < 5mm = moderate; > 5 mm = severe. Bleeding on probing was measured based on visual assessment of bleeding after insertion of a probe into the base of the sulcus or pocket (0 = normal appearing gingiva, no bleeding upon probing; 1 = no color or contour changes, but bleeding upon probing; 2 = bleeding on probing, color change (reddening), no edema; 3 = bleeding on probing, color change, mild inflammatory edema; 4 = bleeding on probing, color change, severe inflammatory edema; 5 = spontaneous bleeding on probing, color change, very severe inflammatory edema with or without ulceration). The tooth score is the average of pocket depth and bleeding on probing for each tooth. The average of pocket depth was multiplied by bleeding on probing to obtain a whole mouth mean pocket score for each animal. Sum of gingivitis score, plaque score, calculus score, and pocket score were used to calculate the OHS.

### Saliva and plaque sample collection

Once scored, plaque (SUP and SUB plaque) and saliva samples were collected for microbiota analysis and the teeth surfaces were cleaned. Saliva samples were collected using two swabs (P-151; DNA Genotek, Ottawa, ON, Canada) per dog according to the manufacturer’s guidelines. Saliva samples were collected where it naturally pools (in the cheek pouch and under the tongue) for 30 s. Swabs were placed into the manufacturer’s tube and shaken vigorously 10 times to thoroughly mix samples. Samples remained in the collection tubes at room temperature during the collection, and then were moved to -20 °C until analysis. Teeth were assessed using a sterile periodontal probe on the gingival margin and sweeping along the base of the crown. SUB and SUP plaque samples were collected from the 4th premolar and 1st molar mandibular teeth and the 4th premolar and 1st molar maxillary teeth. Plaque samples were placed into sterile 2.0 ml cryovials (CryoELITE™, Wheaton™, Millville, NJ, USA) and immediately placed on dry ice until storage at -80 °C, where they were stored until analysis.

### Microbiota analysis

 Total DNA from saliva and plaque samples were extracted using Mo-Bio PowerSoil Kits (MO BIO Laboratories, Inc., Carlsbad, CA, USA), followed by quantification of extracted DNA using a Qubit 3.0 Fluorometer (Life Technologies, Grand Island, NY, USA). Quality of extracted DNA was assessed by electrophoresis using agarose gels (E-Gel EX Gel 1 %; Invitrogen, Carlsbad, CA). Bacterial 16 S rRNA gene amplicons of 252 bp from the V4 region were generated using a Fluidigm Access Array (Fluidigm Corporation, South San Francisco, CA, USA) with Roche High Fidelity Fast Start Kit (Roche, Indianapolis, IN, USA). The primers 515F (5′-GTGCCAGCMGCCGCGGTAA-3′) and 806R (5′-GGACTACHVGGGTWTCTAAT-3′) that target the 252 bp-fragment of V4 region were used for amplification (primers synthesized by IDT Corporation, Coralville, IA, USA; [70]). Quality of the amplicons was assessed using a Fragment Analyzer (Advanced Analytics, Ames, IA, USA) followed by amplicon size selection using electrophoresis and a Qiagen Gel Purification Kit (Qiagen, Valencia, CA, USA). The appropriate profile and average size of purified amplicons were then confirmed using a Bioanalyzer (Agilent Technologies, Santa Clara, CA, USA). Amplicons were sequenced using the Illumina sequencing platform on a MiSeq using v3 reagents (Illumina Inc., San Diego, CA, USA) at the W. M. Keck Center for Biotechnology at the University of Illinois.

Quantitative Insights Into Microbial Ecology (QIIME 2 2018.8; [71]) was used to process the sequence data. Sequence data with quality value ≥ 20 derived from the sequencing process were demultiplexed. Sequences were clustered into operational taxonomic units (OTU) using UCLUST [72] through an open-reference OTU picking strategy against the Greengenes 13_8 reference database [73] with a 97 % similarity threshold. Singletons and OTU that had < 0.01 % of the total observation were discarded. α-diversity was estimated using observed OTU. β-diversity was calculated using weighted and unweighted UniFrac [74] distance measures and presented with principal coordinates analysis plots.

### Statistical analysis

All data were analyzed using SAS (version 9.4, SAS Institute, Cary, NC) using the Mixed Models procedure with dog being considered a random effect, and habitat was considered a fixed effect. Data normality was checked using the univariate procedure and Shapiro-Wilk statistic, with log transformation being used when normal distribution was lacking. If after the logarithmic transformation of the data, the data did not reach normality, the data were analyzed using the npar1way procedure and Wilcoxon statistic. Correlation coefficients were calculated using the Pearson correlation coefficients. Data were reported as means with *p* < 0.05 considered significant. Linear discriminant analysis effect size (LEfSe) [75] was used to evaluate the genetic sequences and to identify genera that were enriched at the various habitats.

## Data Availability

All sequence data are available at the NCBI sequence read archive (http://www.ncbi.nlm.nih.gov/Traces/sra/) under accession number SUB8142132.

## Abbreviations

BOP: Bleeding on probing
OHS: Oral health score
PD: Pocket depth
SAL: Salivary
SUB: Subgingival plaque
SUP: Supragingival plaque
LEfSe: Linear discriminant analysis effect size
LDA: Linear discriminant analysis
