# Correction to: Microbiota populations in supragingival plaque, subgingival plaque, and saliva habitats of adult dogs

**DOI:** 10.1186/s42523-021-00102-7

**Published:** 2021-06-03

**Authors:** Patrícia M. Oba, Meredith Q. Carroll, Celeste Alexander, Helen Valentine, Amy J. Somrak, Stephanie C. J. Keating, Adrianna M. Sage, Kelly S. Swanson

**Affiliations:** 1 Department of Animal Sciences, University of Illinois at Urbana-Champaign, 1207 West Gregory Drive, 162 Animal Sciences Laboratory, Urbana, IL 61801 USA; 2 Division of Nutritional Sciences, University of Illinois at Urbana-Champaign, Urbana, IL 61801 USA; 3 Division of Animal Resources, University of Illinois at Urbana-Champaign, Urbana, IL 61801 USA; 4 Department of Veterinary Clinical Medicine, College of Veterinary Medicine, University of Illinois, Urbana, IL 61801 USA; 5 Department of Surgical Sciences, School of Veterinary Medicine, University of Wisconsin – Madison, 2015 Linden Dr, Madison, WI 53706 USA

**Correction to: Anim Microbiome 3, 38 (2021)**

**https://doi.org/10.1186/s42523-021-00100-9**

During the publication process of this article [1] two errors were introduced:

1. An old version of Fig. 2 was used which did not contain the legend. The updated version of Fig. 2 is available in this correction article.
2. An incorrect affiliation was used for Dr. Sage. The correct affiliation is: Department of Surgical Sciences, School of Veterinary Medicine, University of Wisconsin – Madison, 2015 Linden Dr., Madison WI 53706

**Fig. 2:**
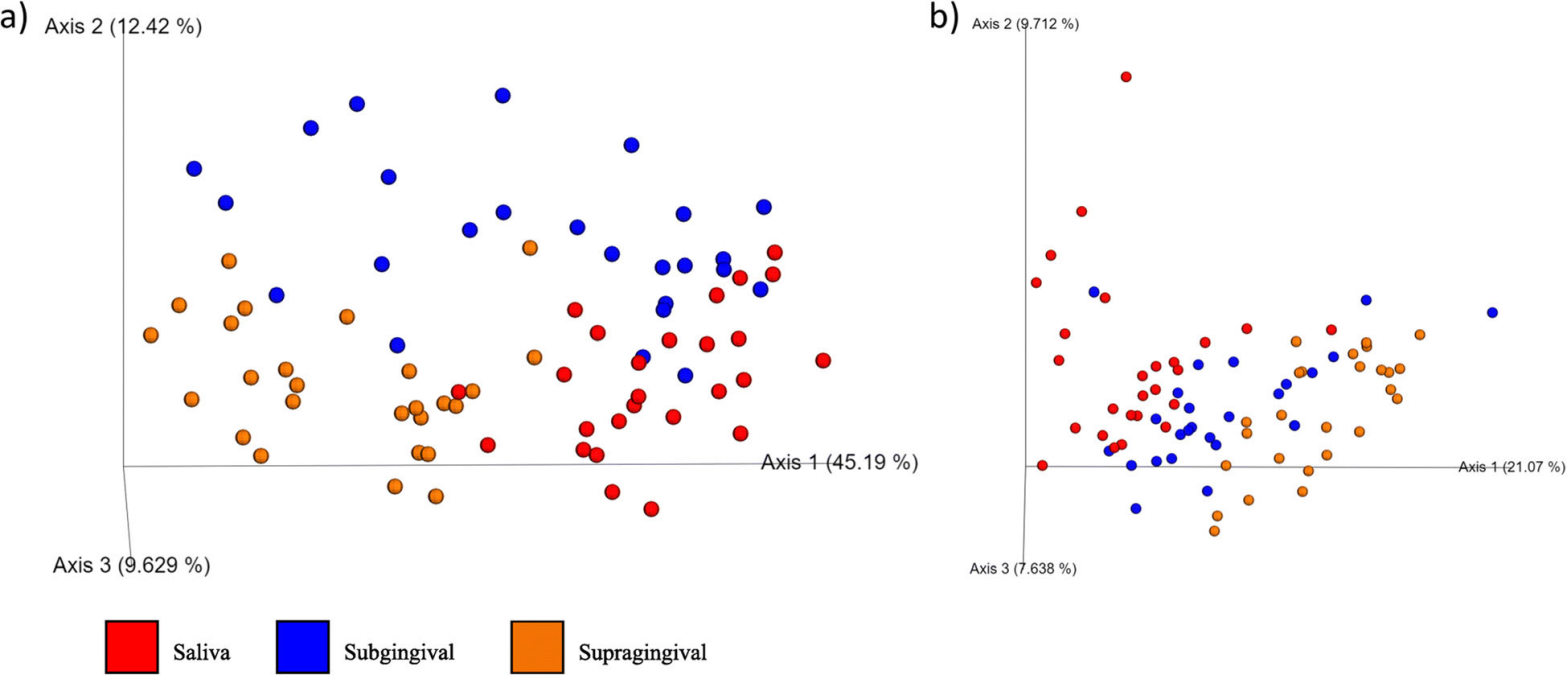
Principal coordinates analysis (PCoA) plots of weighted (**a**) and unweighted (**b**) UniFrac distances of oral microbial communities performed on the 97% OTU abundance matrix using QIIME

The original publication has been updated.
